# Supplementary material for: Sequential Federated Analysis of Early Outbreak Data Applied to Incubation Period Estimation
Source: Epidemics. Author manuscript; Available in PMC 2026 Oct 30. (PMC7619655; doi:10.1016/j.epidem.2026.100890)
Supplement: Supplementary file 1 [file EMS218500-supplement-Highlights.docx]

**Sequential Federated Analysis of Early Outbreak Data Applied to Incubation Period Estimation**

**Highlights**

- Early outbreaks require fast data analyses across multiple locations
- Standard privacy-preserving methods may not comply with severe local restrictions
- Sequential federated analysis requires neither device access nor data sharing
- Epidemiological estimates from sequential federated analysis remain of high quality
- Sequential federated analysis is flexible and easy to deploy during early outbreaks
