# Supplementary material for: Sequential Federated Analysis of Early Outbreak Data Applied to Incubation Period Estimation
Source: Epidemics. Author manuscript; Available in PMC 2026 Oct 30. (PMC7619655; doi:10.1016/j.epidem.2026.100890)
Supplement: Supplementary file 2 [file EMS218500-supplement-Supplement.docx]

**Supplement**

Sequential Federated Analysis of Early Outbreak Data Applied to Incubation Periods Estimation

Simon Busch-Moreno^1^ & Moritz U.G. Kraemer^1,2^

**Affiliations**

1. Department of Biology, University of Oxford, Oxford, UK

2. Pandemic Sciences Institute, University of Oxford, UK

The present supplement includes further justification for the use of gamma sampling distributions and plots with additional summaries of distributions estimated from simulated Mpox data, publicly available Influenza A H7N9, and corona virus disease 2019 COVID-19. Plots include Gamma distributions sumamries with average parameter estimates, and forestplots containing site-level estimates summaries.

To further justify the choice of sampling distribution (Gamma), we emphasise that –theoretically speaking– a Gamma distribution models the sum of *k* i.i.d. exponential random variables, which corresponds to the waiting time until the *k*-th event in a Poisson process (for proof see: Ross, 2019). Lacking exact time counts to model events via a Poisson process, and assuming the incubation process as continuous in time, the Gamma distribution is a theoretically optimal choice. Although other distributions (e.g. log-normal or Weibull) can also provide appropriate results. However, for the sake of simplicity, as our focus is purely on the application of estimation methods for a sequential federated analysis approach, we will exclusively use Gamma sampling distributions. We note, nonetheless, that the methods we present here are agnostic to sampling distributions and in more detailed applications they could eventually incorporate and compare models using different sampling distributions (e.g. log-normal, Weibull, Gamma, etc.).

Presently, we will focus on sequential sampling methods that can preserve Hamiltonian Monte Carlo (HMC) sampling, a variant of Markov Chain Mote Carlo (MCMC) sampling. HMC usually provides more efficient sampling of high dimensional spaces, allowing for more diverse models, plus more informative convergence diagnostics for a better assessment of model sampling and posteriors (Betancourt, 2017). We will introduce two approaches: 1) Prior updates based on a multi-variate normal (MvN) approximation of the joint posterior distributions. This method requires sampling data locally at an initial site (e.g. lab, hospital, etc.) and sharing the posterior distribution of relevant parameters, which will be used to generate the MvN approximation to define the prior distributions at a new site, this new site follows the same procedure with a third site and so on and so forth (for a detailed explanation of Bayesian updates see Kruschke, 2015; McElreath, 2020). 2) Meta-analysis via a Bayesian hierarchical model. This method requires sharing posterior summaries, in particular containing parameters’ posterior means and standard-deviations, which will be used as effect-sizes and errors within the meta-analysis model (for more details on Bayesian meta-analysis see Gelman et al., 2013; Harrer et al., 2021).

Note that site-level estimtes are all named effect-sizes for consistency, but they should be interprested in different ways. Direct Sampling effect-sizes refer to the site means $\mu$ estimated from a whole data hierarchical model (i.e. varying parameters). Meta-analysis effect-sizes refer to the meta-analysis hierarchical model site-by-site estimated means $\mu$ ($\theta_{n}$ in the meta-anaysis hierarchical model). MvN-approx. effect-sizes refer to the $\mu$ estimates from a locally sampled fixed model, where models after the firstly smapled receive $\mu_{n-1}$ information to parametrise priors.

Note Figure S4 panel B, where MvN-approx. approaches better results for G1 and G2, closer to the original study, probably because artificial data chunking has biased the estimates of the other two approaches (without disregarding the possibility of biases present in the original study and our approach due to sampling via a fixed model). We do not expect this effect when data are naturally generated from different sources, which is evidenced by Figure S6, where effect sizes, though differently patterned, remain consistent across approaches (panels A to C), notwithstanding the difficulty of sampling from very low sample-size sites.

Another noteworthy pattern in present plots is the very low uncertainty expressed by Direct Sampling (see Figures S1, S3 and S5, Panels A), which may be due to highly constraining priors. In real situations, less informative priors may be required after prior sensitivity analysis. Oppositely, Meta-analysis hierarchical models induce high uncertainty (see Figures S1, S3 and S5, Panels B), probably due to being over-parametrised (i.e. a much higher number of parameters than datapoints). Even so, they manage to recover estimates with good accuracy.

*S1. Additional Mpox Simulations Summaries*

**Figure S1**. Mpox Simulations PDF summaries. Gamma distributions are computed as $f(x|\alpha,\beta)$ where $f$ is the PDF of the Gamma distribution and $\alpha$ and $\beta$ are shape and rate (their posterior means are shown in plots). The grey shadow indicates the 90% distribution highest density interval (HDI). Dashed, dotted and das-dotted line indicate mean, median and mode of the Gamma distribution respectively, their respective HDIs in brackets.

**Figure S2**. Mpox Simulations Gamma mean ($\mu$) summaries. **A**: Direct Sampling effect-sizes refer to the site means $\mu$ estimated from a whole data hierarchical model (i.e. varying parameters). **B**: MvN-approx. effect-sizes refer to the $\mu$ estimates from a locally sampled fixed model, where models after the firstly smapled receive $\mu_{n-1}$ information to parametrise priors. **C**: Meta-analysis effect-sizes refer to the meta-analysis hierarchical model site-by-site estimated means $\mu$ ($\theta_{n}$ in the meta-anaysis hierarchical model).

*S2. Additional H7N9 Summaries*

**Figure S3**. H7N9 Analysis PDF summaries. Gamma distributions are computed as $f(x|\alpha,\beta)$ where $f$ is the PDF of the Gamma distribution and $\alpha$ and $\beta$ are shape and rate (their posterior means are shown in plots). The grey shadow indicates the 90% distribution highest density interval (HDI). Dashed, dotted and das-dotted line indicate mean, median and mode of the Gamma distribution respectively, their respective HDIs in brackets.

**Figure S4**. H7N9 Analysis Gamma mean ($\mu$) summaries. $\mu_{1}$ and $\mu_{2}$ corrspond to the group 1 and group 2 (G1 and G2) from H7N9 models, where G1 are reovered patients and G2 are fatal cases. **A**: Direct Sampling effect-sizes refer to the site means $\mu$ estimated from a whole data hierarchical model (i.e. varying parameters). **B**: MvN-approx. effect-sizes refer to the $\mu$ estimates from a locally sampled fixed model, where models after the firstly smapled receive $\mu_{n-1}$ information to parametrise priors. **B**: Meta-analysis effect-sizes refer to the meta-analysis hierarchical model site-by-site estimated means $\mu$ ($\theta_{n}$ in the meta-anaysis hierarchical model).

*S3. Additional COVID-19 Summaries*

**Figure S5**. COVID-19 Analysis PDF summaries. Gamma distributions are computed as $f(x|\alpha,\beta)$ where $f$ is the PDF of the Gamma distribution and $\alpha$ and $\beta$ are shape and rate (their posterior means are shown in plots). The grey shadow indicates the 90% distribution highest density interval (HDI). Dashed, dotted and das-dotted line indicate mean, median and mode of the Gamma distribution respectively, their respective HDIs in brackets.

**Figure S6**. COVID-19 Analysis Gamma mean (μ) summaries. **A**: Direct Sampling effect-sizes refer to the site means $\mu$ estimated from a whole data hierarchical model (i.e. varying parameters). **B**: MvN-approx. effect-sizes refer to the $\mu$ estimates from a locally sampled fixed model, where models after the firstly smapled receive $\mu_{n-1}$ information to parametrise priors. Note how sites with very low sample-size [11-23] pull the estimate upwards, despite the strong influence of larger sample-size sites [1-6]. **C**: Meta-analysis effect-sizes refer to the meta-analysis hierarchical model site-by-site estimated means $\mu$ ($\theta_{n}$ in the meta-anaysis hierarchical model).
