# Supplementary material for: Sequential Federated Analysis of Early Outbreak Data Applied to Incubation Period Estimation
Source: Epidemics. Author manuscript; Available in PMC 2026 Oct 30. (PMC7619655; doi:10.1016/j.epidem.2026.100890)
Supplement: Supplementary file 3 [file EMS218500-supplement-Title.docx]

**Sequential Federated Analysis of Early Outbreak Data Applied to Incubation Period Estimation**

Simon Busch-Moreno^1^ & Moritz U.G. Kraemer^1,2^

**Affiliations**

1. Department of Biology, University of Oxford, Oxford, UK

2. Pandemic Sciences Institute, University of Oxford, UK

**Corresponding author:** simon.buschmoreno@ biology.ox.ac.uk

**Abstract**

Early outbreak data analysis is critical for informing about their potential impact and interventions. However, data obtained early in outbreaks are often sensitive and subject to strict privacy restrictions. Thus, federated analysis, which implies decentralised collaborative analysis where no raw data sharing is required, emerged as an attractive paradigm to solve issues around data privacy and confidentiality. In the present study, we propose two approaches which require neither data sharing nor direct communication between devices/servers. The first approach approximates the joint posterior distributions via a multivariate normal distribution and uses this information to update prior distributions sequentially. The second approach uses summaries from parameters’ posteriors obtained locally at different locations (sites) to perform a meta-analysis via a hierarchical model. We test these models on simulated and on real outbreak data to estimate the incubation period of multiple infectious diseases. Results indicate that both approaches can recover incubation period parameters accurately, but they present different inferential advantages. While the approximation approach permits to work with full posterior distributions, thus providing a better quantification of uncertainty; the meta-analysis approach allows for an explicit hierarchical structure, which can make some parameters more interpretable. We provide a framework for federated analysis of early outbreak data where the public health contexts are complex.

**Keywords**: *Collaborative data analysis; Bayesian statistics; approximations; incubation period; infectious disease analysis*

**Acknowledgements**

We thank members of PyMC Discourse who kindly answered our questions regarding federated analysis and posterior to prior updating methods. Many thanks to Jim Sheldon for his valuable input on system management. For the completion of this study all the following software packages were essential: for Bayesian sampling and statistics we use PyMC experimental (PyMC developers, 2024), PyMC (Abril-Pla et al., 2023), and ArviZ (Kumar et al., 2019), for numerical calculation support we use NumPy (Harris et al., 2020) and SciPy (Virtanen et al., 2020), for data wrangling we use pandas (The pandas development team, 2024), and for plots we use Matplotlib (Hunter, 2007).

**Authors Contributions**

Contributions to the study were as follows. S.B.-M.: conceptualisation, software, statistical methodology, analysis, writing. M.U.G.K.: conceptualization, administration, revision.

**Data and Code Availability**

All data and scripts are publicly available at: <https://github.com/kraemerlab/Bayesian_approaches_strict_privacy>

**Funding Sources**

M.U.G.K. acknowledges funding from The Rockefeller Foundation (PC-2022-POP-005), Health AI Programme from Google.org, the Oxford Martin School Programmes in Pandemic Genomics & Digital Pandemic Preparedness, European Union's Horizon Europe programme projects MOOD (#874850) and E4Warning (#101086640), Wellcome Trust grants 303666/Z/23/Z, 226052/Z/22/Z & 228186/Z/23/Z, the United Kingdom Research and Innovation (#APP8583), the Medical Research Foundation (MRF-RG-ICCH-2022-100069), UK International Development (301542-403), the Bill & Melinda Gates Foundation (INV-063472) and Novo Nordisk Foundation (NNF24OC0094346). The contents of this publication are the sole responsibility of the authors and do not necessarily reflect the views of the European Commission or the other funders.

**Declaration of interests**

None.
